# Expressing the Big Picture

**DOI:** 10.1371/journal.pbio.0030105

**Published:** 2005-03-15

**Authors:** Hemai Parthasarathy

## Abstract

A recent conference on neuroesthetics brought artists and scientists together to study empathy

The Fourth International Conference on Neuroesthetics was not a large event, but it was an unusual one. Held on a single day in the basement auditorium of the Berkeley Art Museum at the University of California at Berkeley, it brought together a typically motley collection of intellectuals who would willingly give over a sunny Saturday to an opportunity to learn from a panel of distinguished speakers. This was not the unusual part. Nor was it unusual that the meeting was touted as an interdisciplinary event, bringing together the best and brightest of different fields. These days, and perhaps it has always been the case, interdisciplinarity is the rule rather than the exception of innovative science.

What set this meeting apart was the fluid progression from art to science, in content as well as in style. The artists were more or less scientific, the scientists more or less artistic. The topic was empathy (“Empathy in the Brain and in Art”)—more particularly, man's (and not just man's) ability to recognize and respond to the expressions of others. What do we respond to in an expression and what are the mechanisms in the brain that underlie these responses? And as the primatologist Frans de Waal (Emory University) highlighted, how much of our empathic natures do we share with our ape cousins?

In a slide presentation of her work and sources of inspiration, portrait photographer Judy Dater clearly captured with great sensitivity an infinite variety of poignant expressions. However, when asked, she could not clearly articulate the choices she had made in posing and photographing her subjects, could not give dimensions to the criteria she was using. In contrast, the performance artist Leonard Pitt had clearly made a science out of expression. His physical demonstrations with Balinese masks, carved into iconic images of happiness, sadness, or anger, gave the audience insight into the variety of subtle expression that could be attributed to the mask with simple postural adjustments. Happiness melted into melancholy, sadness into ennui. “It's not about moving,” he observed, “it's about not moving.”

The psychologist Paul Ekman (University of California at San Francisco) brought the official stamp of academia to his science of expression, documenting in the language of training-dependent effects on recognition the subtle range of expressions and microexpressions we can identify. For a practical example, he showed a clip from testimony in the O. J. Simpson trial of a moment in which the infamous “houseguest” Kato Kaelin was caught out in a lie. A fleeting hostile look crossed his otherwise carefully schooled features: invisible until pointed out, unmistakable after.

Where the artist and psychologist show us the richness of the human behavioral repertoire, the neuroscientist tries to break behaviors down into manageable, testable predictions of the associated brain activity. In contrast to the feasts of expression presented by other speakers, the faces representative of basic emotions used by the cognitive neuroscientist Ray Dolan (University College London) to study the neural activity engendered by expressions seemed almost too caricatured to be meaningful. But Dolan, introducing his subject through the portraiture of American colonial artist Gilbert Stewart, deconstructed the information we derive from the expressions of others into five categories—familiarity, identity, emotion, intentionality, and character—and was able to describe neural activity associated with carefully constructed experiments to probe each of these facets.

The Fourth International Conference on Neuroesthetics, "Empathy in the Brain and in Art," took place on 15 January 2005 at the University of California at Berkeley. Further information can be found at http://plaisir.berkeley.edu/.

Physiologist Vittorio Gallese (University of Parma) prompted many nods of satisfaction from the audience with his findings of activity in areas of the brain controlling movement when people simply watched the actions of others (see also the Research Article by Iacoboni et al. in this issue of *PLoS Biology* [DOI: 10.1371/journal. pbio.0030079 ]). Susan Langer, in her book *Mind: An Essay on Human Feeling*, has defined empathy as the direct physical reaction inherent in the perception of others, an *involuntary* breach of individual separateness, and to see the neural resonance, to see that the same activity patterns were being recreated in actor and observer, was to give substance to the intuition of empathy.

Themed meetings, particularly when the theme does not conform to one discipline, are hard to pull off. It can be nearly impossible to convince successful professionals on the lecture circuit to modify the presentation of their own work to support such a theme. In that respect, this meeting was no different from many—some speakers were hard-pressed to conform to the theme, and it is not clear that many attendees learned information of practical value to their work from speakers across disciplines.

However, it is not often that scientists have the luxury of stepping back and appreciating the context of their work in quite this way. It is not, for instance, usually appropriate to begin a paper on an apoptotic signaling pathway with a philosophical digression into the nature of Death. The abstract dimensions that the visual neuroscientist Alice O'Toole (University of Texas at Dallas) gave to facial characteristics are supposed to shed light on how we instantaneously recognize the friend we have not seen in 30 years. The electrophysiological signals in the brain that neurophysiologist Aina Puce (West Virginia University) described when we view simple movements is ultimately meant to explain how we identify with the subtle shrugging of shoulders that can transmute insouciance into insecurity.

By reducing the problem to its simplest, most controlled form, scientists hope to shed light on the complexities of life. Auditory physiologists are supposed to tell us how we hear. And yet it will be a long time before they can explain “music heard so deeply that it is not heard at all, but you are the music while the music lasts” (T. S. Eliot, as quoted by the conference organizer, Semir Zeki [University College London]). But the richness of the goal makes the journey all the more rewarding.

